# Genome Sequencing and Analysis of *Thraustochytriidae* sp. SZU445 Provides Novel Insights into the Polyunsaturated Fatty Acid Biosynthesis Pathway

**DOI:** 10.3390/md18020118

**Published:** 2020-02-18

**Authors:** Xingyu Zhu, Shuangfei Li, Liangxu Liu, Siting Li, Yanqing Luo, Chuhan Lv, Boyu Wang, Christopher H. K. Cheng, Huapu Chen, Xuewei Yang

**Affiliations:** 1 Guangdong Technology Research Center for Marine Algal Bioengineering, Guangdong Key Laboratory of Plant Epigenetics, College of Life Sciences and Oceanography, Shenzhen University, Shenzhen 518060, China; staruny9562@163.com (X.Z.); sfli@szu.edu.cn (S.L.); liangxuliu@icloud.com (L.L.); siting.li@aut.ac.nz (S.L.); lyanqingl87@163.com (Y.L.); LVCHU0510@163.com (C.L.); 17612760955@163.com (B.W.); 2 Shenzhen Key Laboratory of Marine Biological Resources and Ecology Environment, Shenzhen Key Laboratory of Microbial Genetic Engineering, College of Life Sciences and Oceanography, Shenzhen University, Shenzhen 518055, China; 3 Longhua Innovation Institute for Biotechnology, Shenzhen University, Shenzhen 518060, China; 4 School of Biomedical Sciences, The Chinese University of Hong Kong, Hong Kong 999077, China; chkcheng@cuhk.edu.hk; 5 Guangdong Research Center on Reproductive Control and Breeding Technology of Indigenous Valuable Fish Species, Fisheries College, Guangdong Ocean University, Zhanjiang 524088, China; chpsysu@hotmail.com

**Keywords:** whole-genome sequencing, docosahexaenoic acid (DHA), polyunsaturated fatty acid, fatty acid synthesis pathway, polyketide synthase pathway

## Abstract

*Thraustochytriidae* sp. have broadly gained attention as a prospective resource for the production of omega-3 fatty acids production in significant quantities. In this study, the whole genome of *Thraustochytriidae* sp. SZU445, which produces high levels of docosapentaenoic acid (DPA) and docosahexaenoic acid (DHA), was sequenced and subjected to protein annotation. The obtained clean reads (63.55 Mb in total) were assembled into 54 contigs and 25 scaffolds, with maximum and minimum lengths of 400 and 0.0054 Mb, respectively. A total of 3513 genes (24.84%) were identified, which could be classified into six pathways and 44 pathway groups, of which 68 genes (1.93%) were involved in lipid metabolism. In the Gene Ontology database, 22,436 genes were annotated as cellular component (8579 genes, 38.24%), molecular function (5236 genes, 23.34%), and biological process (8621 genes, 38.42%). Four enzymes corresponding to the classic fatty acid synthase (FAS) pathway and three enzymes corresponding to the classic polyketide synthase (PKS) pathway were identified in *Thraustochytriidae* sp. SZU445. Although PKS pathway-associated dehydratase and isomerase enzymes were not detected in *Thraustochytriidae* sp. SZU445, a putative DHA- and DPA-specific fatty acid pathway was identified.

## 1. Introduction

Long-chain polyunsaturated fatty acids (LC-PUFAs), such as docosahexaenoic acid (DHA, 22:6n-3), eicosapentaenoic acid (EPA, 20:5n-3), and arachidonic acid (ARA, 20:4n-6), have gained increasing attention because of their potential health benefits to humans. These benefits have been demonstrated in a number of previous studies, and include the proper development of infant brain and eyes [1,2]; cardiovascular disease prevention [3]; antioxidative, anti-inflammatory [4] as well as anti-cancer properties [5], and the prevention of Alzheimer’s disease [6]. Currently, ocean fish are the primary PUFA production source. However, because of the drawbacks of a fishy odor, the accumulation of contaminants, and declining fish stock, it is necessary to identify other sustainable and relatively safer alternative sources of PUFA [7].

Thraustochytrids, a single-celled eukaryotic marine protist belonging to the class Labyrinthulomycetes, can accumulate large amounts of DHA, resulting in these organisms having attracted a great deal of scientific and industrial interest. Research has shown that some thraustochytrid strains can be cultivated to yield high biomasses containing substantial quantities of lipids abundant in PUFAs [8]. There is an abundance of research on the optimization of fermentation parameters in terms of salinity [9], pH, temperature [10], and cultivation medium [11] for high DHA production. Besides, metabolic engineering is also used as a promising approach to promote DHA productivity. Recent research has indicated that DHA is synthesized by two distinct pathways in thraustochytrid: the fatty acid synthase (FAS) pathway and the polyketide synthase (PKS) pathway [12]. The standard FAS pathway synthesizes fatty acids through a series of elongase- and desaturase-catalyzed reactions. Delta-4 desaturase, delta-5 desaturase, and delta-12 desaturase have been reported in *Thraustochytrium aureum* ATCC 34304 [13,14,15], and delta-5 elongase, delta-6 elongase, and delta-9 elongase have also been successfully identified in some thraustochytrid strains [16,17]. Fatty acids are synthesized through the PKS pathway via highly repetitive cycles of four reactions, including condensation by ketoacyl synthase (KS), ketoreduction (KR), dehydration, and enoyl reduction (ER). Meesapyodsuk and Qiu identified three large subunits of a type I PKS-like PUFA synthase in *Thraustochytrium* sp. 26185 [18]. Nevertheless, to date, the exact biosynthetic mechanism of DHA in *Thraustochytrid* species remains unknown.

De novo genome assembly enables functional genomics research in species whose genetic information is limited, especially in many “non-model” organisms. However, for the *Thraustochytrids* species, the available genomic information is limited. Ye et al. reconstructed a genome-scale metabolic map of *Schizochytrium limacinum* SR21 and found that the biosynthesis of DHA via the PKS pathway requires abundant acetyl-CoA and NADPH, and that 30 genes were predicted as potential targets for DHA overproduction [19]. Comparative genomic analysis revealed that both the FAS and PKS pathways of PUFA production were incomplete and that secreted carbohydrate-active enzymes were remarkably consumed in the genomes of two newly isolated *Thraustochytrids* strains, Mn4 and SW8 [20]. Genome sequence and analysis of *Thraustochytrium* sp. 26185 also showed that both aerobic and anaerobic pathways are present in this strain. However, an essential gene encoding stearate delta-9 desaturase was missing in the aerobic pathway.

*Thraustochytriidae* sp. SZU445 is a promising DHA-producing candidate with DHA production levels up to 45% of the dry cell weight. In this study, the high-quality genome assembly of *Thraustochytriidae* sp. SZU445 is reported using combined second- and third-generation sequencing strategies. The comprehensive genomic analyses provide new insight into PUFA biosynthesis and molecular machinery, laying a framework for future genetic studies and the biotechnological utility of oleaginous microorganisms.

## 2. Results

### 2.1. Genome Sequencing and De Novo Assembly

**Figure 1:**
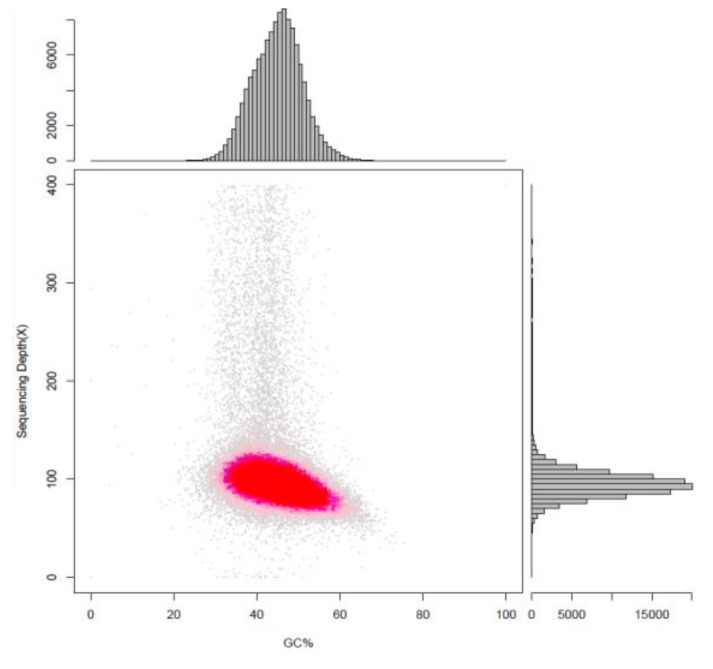
Statistical analysis of the GC content and depth correlation analysis of *Thraustochytriidae* sp. SZU445. The abscissa is the GC content, and the ordinate is the average depth. The scatter plot shows a shape that approximates a Poisson distribution and shows that sequencing data have low GC bias.

**Figure 2:**
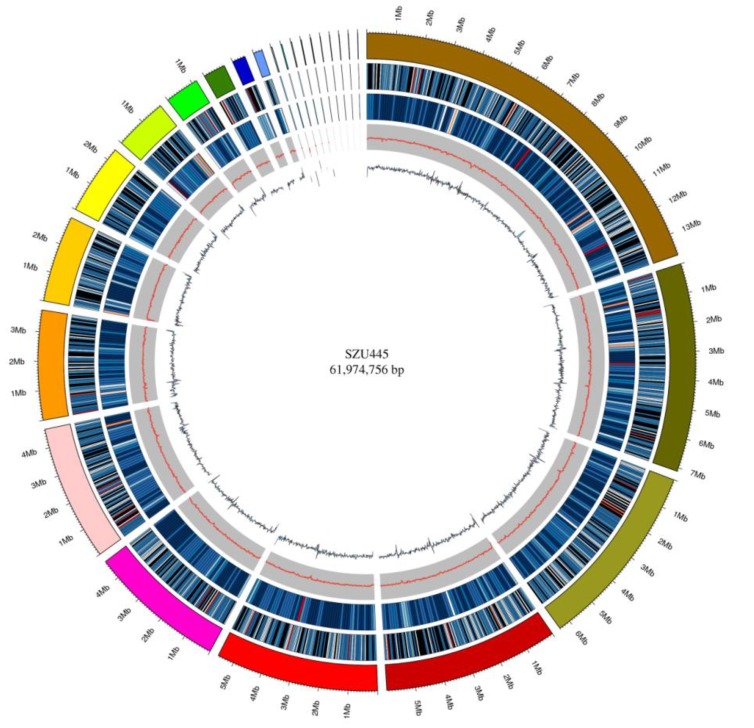
Genomic circle diagram of *Thraustochytriidae* sp. SZU445. From the outer to the inner rings: Genome (sorted by length), gene density (gene number in 50,000 bp nonoverlapping windows), ncRNA density (ncRNA number in 100,000 bp nonoverlapping windows), GC (GC rate in 20,000 bp nonoverlapping windows), GC_skew (GC skew in 20,000 bp nonoverlapping windows).

**Figure 3:**
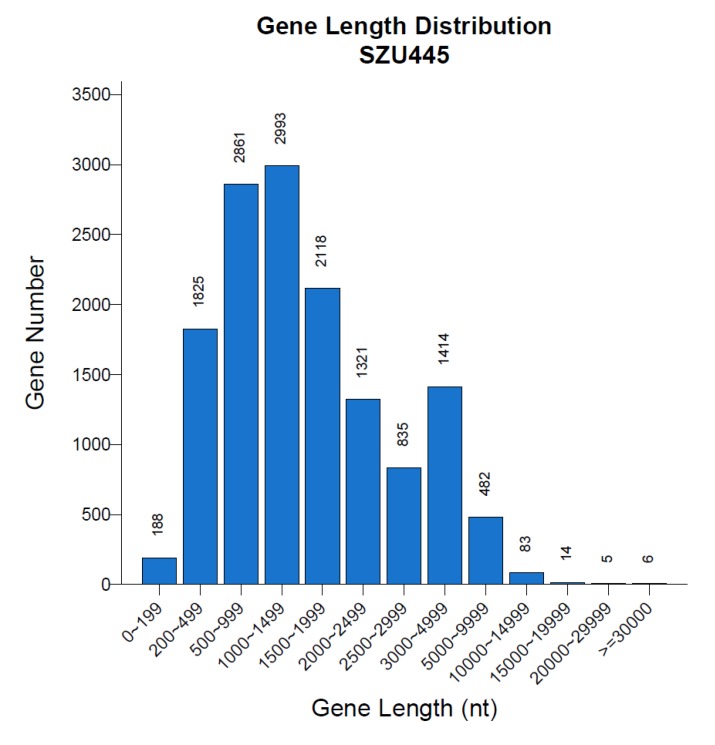
Gene length distribution of *Thraustochytriidae* sp. SZU445. The abscissa is the length of the gene, and the ordinate is the number of genes corresponding to the length of the gene.

A total of 64.71 Mb raw data were generated by PacBio platform (BGI, Shenzhen, China) and MGI2000 platform (MGI, Shenzhen, China). After shortening the adaptor sequences and filtering out low-quality data, 63.55 Mb of clean data was obtained. The PacBio platform was then used to generate high-quality subreads. From 1,055,214 subreads, 11,245,510,279 bp were obtained with 25 scaffolds and 54 contigs generated after sequence assembly (Table 1). The N50 values of the scaffolds and contigs were 5,997,638 bp and 2,552,134 bp, respectively. The GC content of the scaffolds and contigs was 45.04%. The Poisson distributions of the GC content and GC depth analysis (Figure 1) suggested that there was no external DNA contamination or sequencing bias, demonstrating that the genome assembly was of high quality. In addition, according to the sequence of the sequenced marine fungus, the (G − C)/(G + C) calculation method was used for GC skew analysis, and based on the results of gene distribution, ncRNA distribution, and annotation, a gene circle was constructed that indicated the distribution of each component in the genome (Figure 2). The genome of *Thraustochytriidae* sp. SZU445 (Table 2) contains approximately 14,145 genes, 18,768 exons, 14,145 CDSs, and 4623 introns. The total length of genes is 26,947,341 bp, with an average length of 1905.08 bp. Among all genes, those with lengths of 200–499 nt and 500–999 nt were drastically more abundant than those of other lengths (Figure 3). Based on the assembly and annotation results, 1106 copies of the noncoding RNA were predicted, which included nearly 493 copies (0.06%) of tRNA, 235 copies (0.64%) of rRNA, and 77 copies (0.01%) of snRNA (Table 3).

### 2.2. Genome Sequence Annotation

All genes of *Thraustochytriidae* sp. SZU445 were aligned to sequences in 18 public databases, including the Carbohydrate-Active enZYmes Database (CAZY), the Transporter Classification Database (TCDB) database, Swiss-Prot, InterPro, GO, KOG/COG, and KEGG (Table 4). The number and percentage of genes annotated by each database are presented in Table 4. Notably, 30.35% of the genes were unidentifiable, the percentage of which was similar to that observed in other species [21,22,23].

**Figure 4:**
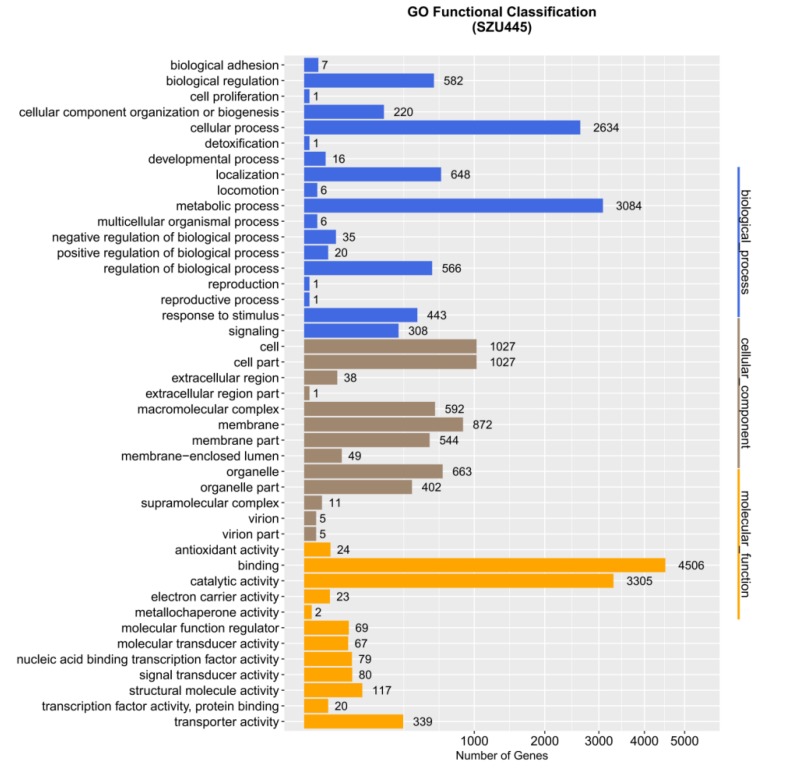
Distribution of GO database functional annotations. The ordinate is the annotation item, and the abscissa is the number of corresponding genes.

The GO database was founded by the Gene Ontology Association in 1988 including three categories: (1) cellular component, used to describe locations, subcellular structures, and macromolecular complexes such as nucleoli, telomeres, and the recognition of the initial complex; (2) molecular function, used to describe the gene functions and gene products, such as binding to carbohydrates or ATP hydrolase activity; and (3) biological process, used to describe the ordered combination of molecular functions to achieve broader biological functions, such as mitosis [24]. Genes are assigned to one or more of these categories depending on the nature of the product. Through the GO database annotation, we speculated the possible functions of genes based on their annotations in different categories. In *Thraustochytrium* sp. SZU445, 22,446 genes were annotated based on the GO database (Figure 4). The majority genes in the biological process category were involved in cellular and metabolic processes, with 3084 and 2634 genes identified, respectively. Simultaneously, only one gene participated in cell proliferation and detoxification in the cellular component category. For the cellular component category, cell (1027 genes), cell part (1027 genes), and membrane (872 genes) were the top three terms. In the molecular function category, the number of genes participating in binding (4506 genes) and catalytic activity (3305 genes) occupied the dominant position. Only one gene related to metallochaperone activity was identified in the molecular function category. According to the literature, fatty acid synthesis-related genes are primarily classified in the metabolic process group of biological process [25,26]. Thus, fatty acid anabolic processes dominated the biological processes of *Thraustochytrium* sp. SZU445.

**Figure 5:**
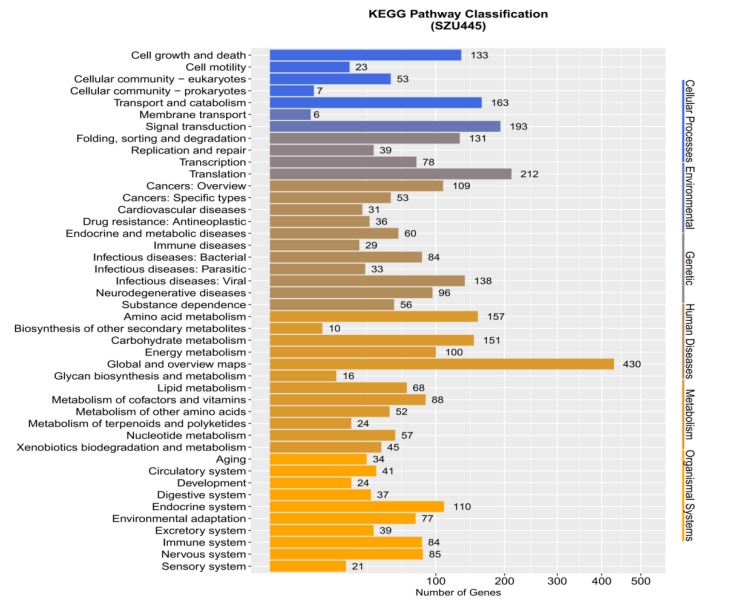
Distribution of KEGG database functional annotations. The ordinate is the annotation item, and the abscissa is the number of corresponding genes.

KEGG is a database of large-scale molecular data generated from molecular-level information—particularly genome sequencing and other high-throughput experimental techniques—to understand the advanced functions and utility of biological systems. The database divides biological pathways into eight categories, each of which has subdivisions. Each subdivision is annotated with an associated gene that is then graphically displayed. Through this database annotation, it is easy to find genes of all annotations related to a specific function. According to the results, 3513 genes were assigned into 6 KEGG classifications (cellular processes, environmental, genetic, human diseases, metabolism, and organismal systems) in *Thraustochytrium* sp. SZU445 (Figure 5). The percentages of genes involved in the 6 KEGG classifications (metabolism and organismal systems, environmental, human diseases, genetic, cellular processes) were 10.84%, 5.65%, 13.13%, 20.66%, 34.10%, and 15.74%, respectively. Among them, the dominant classification was genes involved in metabolism pathways (1198 genes). The metabolism classification can also be divided into 12 subclassifications (metabolism of amino acid, biosynthesis of other secondary, carbohydrate, energy, lipid, cofactors and vitamins, other amino acids, terpenoids and polyketides, nucleotide, global and overview maps, glycan biosynthesis and metabolism, xenobiotic biodegradation, and metabolism). Sixty-eight genes participated in lipid metabolism, which was fifth among the remaining subclassifications. This result provided the foundation for further gene analysis of fatty acid metabolism. In particular, the number of genes involved in global and overview maps (430 genes) was the largest among all subclassifications and classifications.

### 2.3. Phylogenetic Analysis of Thraustochytriidae sp. SZU445

**Figure 6:**
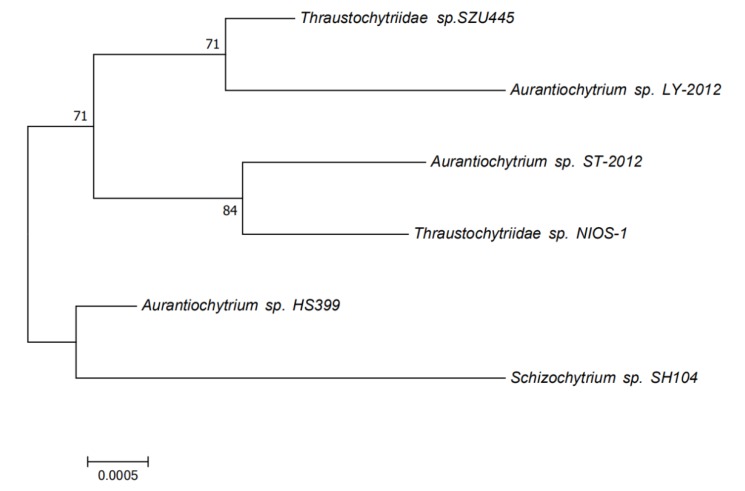
The phylogenetic tree produced using the neighbor-joining method analysis. The evolutionary history was inferred using the neighbor-joining method. The optimal tree with the sum of branch length = 0.01309240 is shown. The percentage of replicate trees in which the associated taxa clustered together in the bootstrap test (1000 replicates) is shown next to the branches. The evolutionary distances were computed using the maximum composite likelihood method and are in the units of the number of base substitutions per site. The analysis involved six nucleotide sequences. Codon positions included were 1st + 2nd + 3rd + noncoding. All ambiguous positions were removed for each sequence pair. There were a total of 1739 positions in the final dataset. Evolutionary analyses were conducted in MEGA7.

Based on the 18S rRNA sequences of the sample Thraustochytriidae sp. SZU445 and the reference strains, we used the MEGA software to construct a phylogenetic tree. The neighbor-joining method was applied for the analysis. As shown in Figure 6, Thraustochytriidae sp. SZU445 was shown to share a common ancestor with all reference strains. The evolutionary position of SZU445 is lower compared with the reference strains, while it is higher than Aurantiochytrium sp. HS399. In terms of kinship, Thraustochytriidae sp. SZU445 showed a higher kinship than Aurantiochytrium sp. LY-2012.

### 2.4. Analysis of Genes Involved in Long-Chain Fatty Acid (LCFA) Biosynthesis

We selected the genes that participated in LCFA biosynthesis from the genome sequencing and protein annotation results in the KEGG database (Table 5). There are two main ways to synthesize LCFAs: The FAS pathway and the PKS pathway.

In *Thraustochytrium* sp. SZU445, FAS (KEGG, EC: 2.3.1.86) (total gene length: 12,443 bp; CDS length: 12,285 bp; number of amino acids: 4147; number of copies: 1) and acyl carrier protein (ACP) (COG, COG ID:COG0236) (total gene length: 429 bp; CDS length: 429 bp; number of amino acids: 143; number of copies: 1) were sequenced and annotated. In the FAS pathway, the acetic acid to long-chain saturated fatty acids (C16:0 and C18:0) step of primary fatty acid biosynthesis is catalyzed by a type I FAS, and long-chain unsaturated fatty acids are generated after a string of desaturation and elongation reactions through desaturases and elongases [27]. FAS is a multifunctional polypeptide of 4147 amino acids that includes four catalytic domains for biochemical reactions: condensation, ketoacyl reduction, hydroxyacyl dehydration, and enoyl reduction [28]. Additionally, it also possesses an ACP domain for binding of the phosphopantetheine prosthetic group to carry an acyl chain. Furthermore, a malonyl-CoA ACP transacylase (MAT) domain, that can catalyze the transformation of malonyl-CoA to malonyl-ACP, was also detected in the enzyme [29]. In the aerobic pathway, the biosynthesis of LCFAs involves a string of desaturation and elongation reactions. Therefore, desaturase and elongase are highly significant in the aerobic FAS pathway. The second half of the FAS pathway can be divided into two subpathways. One subpathway starts with C18:3 (9,12,15), the biosynthesis of which involves the delta-6 desaturation of C18:3 (9,12,15) to C18:4 (6,9,12,15), the elongation of C18:4 (6,9,12,15) to C20:4 (8,11,14,17), and the delta-5 desaturation of C20:4 (8,11,14,17) to C20:5 (5,8,11,14,17) followed by another elongation of C20:5 (5,8,11,14,17) to C22:5 (7,10,13,16,19) and a final delta-4 desaturation of C22:5 (7,10,13,16,19) to C22:6 (4,7,10,13,16,19). The same enzymes are involved in both the first and second pathways. In *Thraustochytrium* sp. SZU445, two desaturases and elongases were detected: delta-4 (KEGG, EC: 1.14.19.17 1.14.18.5) (total gene length: 1471 bp; CDS length: 1167 bp; number of amino acids: 388; number of copies: 1), delta-9 (KEGG, EC: 1.14.19.18) (total gene length: 1601 bp; CDS length: 1601 bp, number of amino acids: 541; number of copies: 1) and elongase (KOG, KOG ID:KOG3072) (total gene length: 1088 bp; CDS length: 1088 bp; number of amino acids: 362; number of copies: 1). These protein products exhibited 100.00%, 95.54%, and 99.87% similarity to the sphingolipid delta-4 desaturase of *Hondaea fermentalgiana* (NCBI accession: GBG25593.1), the delta-9 desaturase of *Hondaea fermentalgiana* (NCBI accession: GBG34675.1), and the elongation of very-long-chain fatty acids protein 6 of *Hondaea fermentalgiana* (NCBI accession: GBG25297.1) [30]. The characterization of this desaturase gene and its function was reported by exogenous expression in yeast [31,32,33]. However, there were only two desaturases (delta-4 and delta-9 desaturase) annotated in *Thraustochytrium* sp. SZU445 that belonged to the classic FAS pathway. The results indicated that a nonclassic FAS pathway may exist in *Thraustochytrium* sp. SZU445 for LC-PUFA synthesis.

PKS has been identified in prokaryotes and eukaryotes [12]. Briefly, fatty acids and polyketides are constructed by repeated decarboxylative Claisen ester condensations of an acyl-CoA starter part with malonyl-CoA units catalyzed by a 3-ketoacyl-synthase (KS). This process often involves an ACP and a (malonyl) acyltransferase (MAT/AT). In fatty acid biosynthesis, beta-oxidation processing typically yields an entirely saturated acyl backbone by ketoreductase (KR), dehydratase (DH), and an enoyl reductase (ER) [34]. An isomerase changes the conformation of the fatty acid chain. KS (KEGG, EC: 2.3.1.179) (total gene length: 1335 bp; CDS length: 1335 bp; number of amino acids: 444; number of copies: 1), KR (KEGG, EC: 1.1.1.-) (total gene length: 1489 bp; CDS length: 1266 bp; number of amino acids: 422; number of copies: 1), and ER (KEGG, EC: 1.3.1.9) (total gene length: 1146 bp; CDS length: 1146 bp; number of amino acids: 382; number of copies: 1) were detected in *Thraustochytrium* sp. SZU445 by genome sequencing and protein annotation. The KS, KR, and ER were highly homologous to the beta-ketoacyl-acyl-carrier-protein synthase II of *Aphanomyces invadans* (NCBI accession: XP_008875515.1), oxidoreductase of “*Candidatus Poribacteria*” bacterium (NCBI accession: RKU36622.1) [35] and tRNA-dihydrouridine(47) synthase NADP+-like of *Hondaea fermentalgiana* (NCBI accession: GBG27734.1) [30] with approximately 94.45%, 95.67%, and 99.00% identity at the amino acid level. These results demonstrate that *Thraustochytrium* sp. SZU445 may possess the PKS pathway but not the classical pathway. Nevertheless, neither the gene nor protein for the DH and isomerase were discovered in *Thraustochytrium* sp. SZU445. We found two enzymes in *Thraustochytrium* sp. SZU445 with functions that were highly similar to those of a DH and an isomerase. These two enzymes were dTDP-glucose 4,6-DH (KEGG, EC: 4.2.1.46) (total gene length: 1134 bp; CDS length: 1134 bp; number of amino acids: 377; number of copies: 1) and delta-3,5-delta-2,4-dienoyl-CoA isomerase (KEGG, EC: 5.3.3.21) (total gene length: 852 bp; CDS length: 852 bp; number of amino acids: 283; number of copies: 1). Both of these proteins were 94.00% and 99.00% homologous to the dTDP-glucose 4,6-DH of *Fistulifera solaris* (NCBI accession: GAX23278.1) [36] and delta-3,5-delta-2,4-dienoyl-CoA isomerase of *Hondaea fermentalgiana* (NCBI accession: GBG25986.1) [30]. Thus, we propose that these two enzymes are the isozymes of DH and isomerase and that they participate in the PKS pathway together with KS, ER, and ER. The specific functions of dTDP-glucose 4,6-DH and delta-3,5-delta-2,4-dienoyl-CoA isomerase are discussed in the next section.

## 3. Discussion

### 3.1. Fatty Acid Synthesis by the FAS Pathway

In the FAS pathway, the synthesis of PUFAs can be divided into two parts [37]. In the first part, palmitic acid (C16:0) is synthesized by the dehydration of acetyl-CoA and malonyl-COA by the FAS complex (NOG, NOG ID: 49.09) [38]. In the second part, palmitic acid (C16:0) is transformed into a very-long-chain (C22) fatty acid by various enzymes. The FAS complex consists of an ACP (COG, COG ID: COG0236) and six enzyme monomers [39]. The six enzyme monomers are acetyl-CoA-ACP transacylase, malonyl-CoA-ACP transacylase, beta-ketoacyl-ACP synthase, beta-ketoacyl-ACP reductase, beta-hydroxyacyl-ACP, DH, enoyl-ACP reductase, and palmitoyl-ACP thioesterase [40]. Then, after a string of desaturation and elongation reactions by delta-9, delta-12, delta-15, delta-6, and delta-4 desaturases [31] and elongases, palmitic acid obtains double bonds, extending the carbon chain and finally generating PUFAs. In *Thraustochytrium* sp. SZU445, FAS (NOG, NOG ID: 49.09) and ACP (COG, COG ID: COG0236) were identified. Therefore, the saturated fatty acids before C18:0 can be synthesized. Interestingly, *Thraustochytrium* sp. SZU445 was not observed to contain delta-12, delta-15, or delta-6 desaturases by whole-genome sequencing and protein annotation; however, it could synthesize PUFAs. A similar phenomenon has also been reported in a number of other studies, where *Arthrospira platensis* was observed to only have 15-*cis*-phytoene desaturase and zeta-carotene desaturase, and *Strongylocentrotus nudus* was observed to only have delta-5, delta-6, and delta-9 desaturases [41,42,43,44]. This result may indicate that the FAS pathway is not the primary pathway responsible for PUFA synthesis. 

### 3.2. Fatty Acid Synthesis by the PKS Pathway

Polyketides, the secondary metabolites that harbor multiple units of ketide groups (-CH_2_-CO-) [45], are synthesized by PKS, which is an enzyme similar to FAS in bacteria [12]. Similar to the FAS pathway, by using ACP as a covalent connection of PUFA synthesis [39,46] (COG, COG ID: COG0236), the PKS pathway proceeds with repeated cycles. The whole cycle includes condensation by using an acyl-ACP and a malonyl-ACP to form a ketoacyl-ACP with KS (KEGG, EC: 2.3.1.179), keto-reduction by converting ketoacyl-ACP to hydroxyacyl-ACP by KR (KEGG, EC: 1.1.1.-), and dehydration by removing a water molecule from hydroxyacyl-ACP. One whole cycle can produce an unsaturated enoyl-ACP, which is then reduced to a saturated acyl chain by ER (KEGG, EC: 1.3.1.9) [47]. However, unlike the FAS pathway, the PKS pathway often neglects steps of the whole cycle, e.g., dehydration and reduction. Therefore, the products of the PKS pathway vary greatly in structure and often contain keto and hydroxyl groups and double bonds. KS, KR, and ER were discovered in *Thraustochytrium* sp. SZU445, but an isomerase and a DH were not identified; however, *Thraustochytrium* sp. SZU445 can also generate PUFAs such as DHA (C22:5) [42,43,44].

### 3.3. Putative Fatty Acid Synthesis Pathway in Thraustochytrium sp. SZU445

Since *Thraustochytrium* sp. SZU445 lacked complete FAS and PKS pathways, we speculated that the synthesis of SZU445 LC-PUFAs involves a combination of the two pathways. According to the whole-genome sequencing and protein annotation results, no DH or isomerase genes were found in *Thraustochytrium* sp. SZU445. However, we identified two enzymes with functions that were highly similar to those of DH and isomerase. The two enzymes are dTDP-glucose 4,6-DH (KEGG, EC: 4.2.1.46) and delta-3,5-delta-2,4-dienoyl-CoA isomerase (KEGG, EC: 5.3.3.21). dTDP-glucose 4,6-DH can catalyze the transformation of dTDP-glucose into dTDP-4-keto-6-deoxyglucose, forming a ketone group [48]. The isomerization of 3-*cis*-octenoyl-CoA to 2-*trans*-octenoyl-CoA can be catalyzed by delta-3,5-delta-2,4-dienoyl-CoA isomerase with specific activity for altering the conformation. Moreover, delta-3,5-delta-2,4-dienoyl-CoA isomerase is indispensable for the beta-oxidation of PUFAs [49]. These two enzymes may be the isoenzymes of DH and isomerase and, hence, perform the same functions.

The protein annotation of *Thraustochytrium* sp. SZU445 revealed that the DH and isomerase of the classic PKS pathway are not present in *Thraustochytrium* sp. SZU445. In addition, only the delta-4 and delta-9 desaturases of the classic FAS pathway were found. Moreover, the fatty acid composition of *Thraustochytrium* sp. SZU445 was analyzed using gas chromatography and shown to primarily include methyl tetradecanoate (C14:0), methyl hexadecanoate (C16:0), DPA, and DHA C20:4 (7,10,13,16). Based on these results, we speculated upon the possible synthesis pathway of long-chain fatty acids in *Thraustochytrium* sp. SZU445.

**Figure 7:**
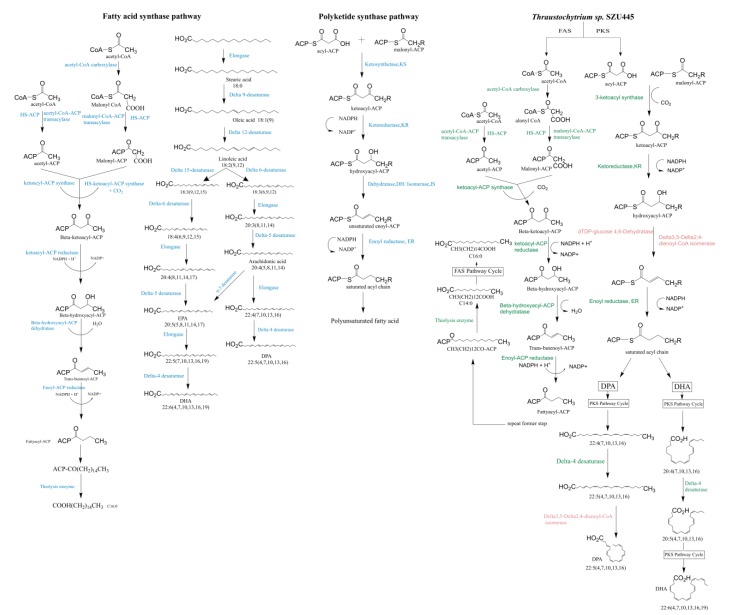
Putative fatty acid synthase pathway of *Thraustochytriidae* sp. SZU445 with the classical fatty acid synthesis pathway and the polyketide synthase pathway. The enzymes colored blue are present in the classical fatty acid synthase (FAS) and polyketide synthase (PKS) pathways. The enzymes colored green are present in *Thraustochytriidae* sp. SZU445 and correspond to the classical FAS and PKS pathways. The enzymes colored red are isozymes of dehydrase and isomerase in the PKS pathway that exist in *Thraustochytriidae* sp. SZU445.

We inferred that long-chain saturated fatty acids of *Thraustochytrium* sp. SZU445, such as methyl hexadecanoate, are produced by the FAS pathway and that long-chain unsaturated fatty acids, such as DHA, are produced by the PKS pathway, with one desaturase participating in this process instead of the desaturation and elongation steps in the FAS pathway (Figure 6). For the long-chain saturated fatty acids, acetyl-CoA and malonyl-CoA are used as substrates to yield methyl tetradecanoate (C14:0) and methyl hexadecanoate (C16:0) by cycling through the FAS pathway six and seven times, respectively. For the long-chain unsaturated fatty acid DPA, C22:4 (7,10,13,16) is generated by cycling through the PKS pathway ten times. C22:5 (4,7,10,13,16) is then converted from C22:4 (7,10,13,16) via delta-4 desaturase. DPA is eventually produced by the activity of delta-3,5-delta-2,4-dienoyl-CoA isomerase (KEGG, EC: 5.3.3.21). In the biosynthesis of DHA, C20:4 (7,10,13,16) is produced by circulating through the PKS pathway nine times. C20:5 (4,7,10,13,16) is formed through the addition of a double bond on the carbon at the fourth position by delta-4 desaturase. Finally, DHA biosynthesis is completed after one additional cycle of the PKS pathway (Figure 7).

## 4. Materials and Methods

### 4.1. Microbes and Cultivation

*Thraustochytrium* sp. SZU445 was isolated from mangroves (22°31′13.044″ N, 113°56′58.560″ E) in the coastal waters of southern China. Because of the results of an initial assessment for DHA production, *Thraustochytrium* sp. SZU445 was selected for further research. Furthermore, *Thraustochytrium* sp. SZU445 was stored in the China General Microbiological Culture Center (CGMCC) under the accession no. 8095. First, the strain was inoculated into M4 liquid medium, which was prepared in 100% filtered natural seawater containing 0.15% peptone, 2% glucose, 0.025% KH_2_PO_4_, and 0.1% yeast extract. Seawater was gathered from Mirs Bay, Shenzhen (22°31′32.632″ N, 114°28′40.185″ E), with a salinity of approximately 30.75–33.19‰. Cultures were incubated for 48 h at a constant temperature of 27 °C and with shaking at 180 rpm. Subsequently, 4% of the culture was used to inoculate fresh M4 medium and incubated for three days under the same growth conditions.

### 4.2. DNA Preparation and Sequencing

The sequencing platform chosen for this subject was a combination of the PacBio (BGI, Shenzhen, China) and MGI2000 (MGI, Shenzhen, China) platforms. Genomic DNA was extracted from a 50 mL fresh cell suspension of *Thraustochytrium* sp. SZU445, which was centrifuged at 8000 rpm for 10 min. The resulting cell pellets were ground to a fine powder in liquid nitrogen, then washed once in 5.0 mL DNA extraction buffer. Subsequently, the pellets were resuspended in 5.0 mL phenol/chloroform/isoamyl alcohol (25:24:1) and centrifuged twice at 9000 rpm for 16 min. The supernatant was then washed in an equal volume of chloroform and centrifuged at 12,000 rpm for 16 min. Genomic DNA was precipitated by adding 2.5 volumes of 100% ethanol and collected by centrifugation at 12,000 rpm for 15 min at 4 °C. The genomic DNA of *Thraustochytrium* sp. SZU445 was sequenced on the PacBio sequencing platform based on third-generation sequencing technologies. Prior to library construction, the concentration, integrity, and purity of the genomic DNA was tested. The concentration was detected using a Qubit fluorometer (Invitrogen, Carlsbad, CA, USA). The purity and integrity of the sample was assessed by agarose gel electrophoresis (agarose gel concentration: 1.2%; voltage: 120 V; electrophoresis time: 50 min). To construct the library for the PacBio platform, 10 μg of genomic DNA was randomly fragmented by g-TUBE (Covaris, Woburn, MA, USA). The fragmented genomic DNA was then processed using an Agencourt AMPure XP-Medium kit (Beckman Coulter, Brea, CA, USA) to obtain fragments with a size of 10 kb. The DNA fragments generated from exonuclease ExoVII (New England Biolabs, Ipswich, MA, USA) digestion and subject to end repair were connected to the hairpin structure at both ends to form a dumbbell structure called SMRTbell. The library for the PacBio sequencing platform was successfully constructed after the purification and sorting of fragments. Qualified library fragments were sequenced after the annealing and binding polymerase steps. The genome sequencing data of *Thraustochytriidae* sp. SZU445 were uploaded to the National Center for Biotechnology Information Search database (NCBI) (BioProject ID: PRJNA579065; BioSample accession: SAMN13135800; SRA accession: PRJNA579065). The method for constructing the library using the MGI2000 platform (MGI, Shenzhen, China) is essentially the same as that described for the PacBio sequencing platform (BGI, Shenzhen, China).

### 4.3. Fragment Assembly and Gene Annotation

Since the raw sequencing data contained low-quality sequences, such as adaptors as well as duplicated and low-quality reads, these reads were filtered to obtain reliable subreads for assembly and guarantee the reliability of the subsequent analysis results. Genome assembly can be divided into the following four parts. (1) Subreads correction: Use Proovread (Version: 2.12; Parameter setting: -t 4 --coverage 60 --mode sr; related website: https://github.com/BioInf-Wuerzburg/proovread) to perform mixed correction on Subread to get highly reliable corrected reads; (2) corrected reads assembly: based on the corrected reads, Celera (Version: 8.3; parameter setting: doTrim_initialQualityBased = 1, doTrim_finalEvidenceBased = 1, doRemoveSpurReads = 1, doRemoveChimericReads = 1, - d properties -U; related website: http://sourceforge.net/projects/wgs-assembler/files/wgs-assembler/wgs-8.3/) and Falcon were each used to generate separate assemblies. Finally, the optimal assembly result was selected; (3) assembly result correction: single-base correction by GATK (Version: v1.6-13; parameter settings: -cluster 2 -window 5 -stand_call_conf 50 -stand_emit_conf 10.0 -dcov 200 MQ0 ≥ 4; related website: http://www.broadinstitute.org/gatk/) for assembly using second-generation small fragment data to obtain highly reliable assembly sequences; (4) scaffold and hole filling: a scaffold was constructed using SSPACE_Basic_v2.0 (Version: v2.0; related website: http://www.baseclear.com/genomics/bioinformatics/basetools/SSPACE) on the assembly results based on the second-generation Illumina large-segment data, and pbjelly2 (Version: 15.8.24; related website: https://sourceforge.net/projects/pb-jelly/) was used to fill holes in the scaffold. After completing the genome assembly, we first constructed the gene model for subsequent gene function annotation. We chose the de novo prediction to build the gene model. The de novo prediction uses the GeneMark-ES software (Version: 4.21, parameter settings: --genemarkes, Related website: http://exon.gatech.edu/). This software is based on Hidden Markov Model and ab initio algorithm to make predictions, and it does not require reference species and reference sequences. It is self-trained. In the assembled sequence, all possible open reading frames (ORF) were predicted as CDSs by the GeneMarkes software. After the prediction was finished, the predicted ORF was translated into the amino acid sequence. Then it was compared with 18 protein databases such as Kyoto Encyclopedia of Genes and Genomes (KEGG) database (Version: 81), Gene Ontology (GO) (Version: releases_2017-09-08), and Swiss-Prot database (Version: release-2017-07) by BLAST to obtain corresponding functional annotation information. Since each sequence alignment result exceeds one, in order to ensure its biological significance, we retain an optimal alignment result as the annotation of the gene. The Eukaryotic Cluster of Orthologous Groups (KOG) and Kyoto Encyclopedia of Genes and Genomes (KEGG) database pathway annotations were implemented using a separate BLAST search against both the KOG (http://www.ncbi.nlm.nih.gov/COG/) and KEGG (https://www.genome.jp/kegg/) databases. The Gene Ontology (GO) and Swiss-Prot annotations were also carried out using the Swiss-Prot database (https://web.expasy.org/docs/swiss-prot_guideline.html) and the GO database (https://www.ebi.ac.uk/ols/ontologies/go), respectively. In addition, the prediction and annotation of kinase domains in the Eukaryotic Protein Kinases and Protein Phosphatases Database (EKPD) were performed by HMMER (v.3.0) using the default parameters and the kinase database (http://ekpd.biocuckoo.org/faq.php). Moreover, for non-coding RNA prediction, the rRNA was predicted by RNAmmer software (Version: 1.2, parameter settings: –s Species –m Type –gff*.rRNA.gff –f*.rRNA.fq, Related website: http://www.cbs.dtu.dk/services/RNAmmer/). The tRNA region and tRNA secondary structure predicted by tRNAscan-SE software (Version: 1.3.1, parameter settings: –Spec_tag(BAOG) –o *. tRNA –f*.tRNA.structure, Related website: http://gtrnadb.ucsc.edu/). SnRNAs was predicted in comparison with Rfam database (Version: 9.1, parameter settings: –p blastn –W 7 –e 1 –v 10000 –b 10000 –m 8 –i subfile –o *.blast.m8, Related website: http://rfam.sanger.ac.uk/) by using the "covariance model" (CMS) of the Infranal software (Version: 1.1.1 (July 2014), parameter settings: default parameter, Related website: http://rfam.sanger.ac.uk/). The default parameters of Infranal software is displayed in Supplementary Materials.

### 4.4. Phylogenetic Analysis

The 18S rRNA was selected as the alignment sequence, and the 18S rRNA sequences of the reference strains were obtained from National Center for Biotechnology Information (NCBI). The blast function of the NCBI was used to compare the similarity of *Thraustochytriidae* sp. SZU445 and those of the reference strains. The NCBI accession numbers of the reference strains are shown in Table 6.

The MEGA software (Version: 7.0.26; algorithm: neighbor-joining method, the maximum composite likelihood method for the evolutionary distances; related website: https://www.megasoftware.net/) was used to construct a phylogenetic tree for *Thraustochytriidae* sp. SZU445 and the reference strains.

## 5. Conclusions

In summary, this research revealed the essential genome features of *Thraustochytriidae* sp. SZU445 and proposed a putative fatty acid synthesis pathway (specifically for DHA and DPA). Our purpose was to identify metabolism-related genes involved in PUFA synthesis in *Thraustochytriidae* sp. SZU445. This study is the first to provide genome information relating to *Thraustochytriidae* sp. SZU445 using a third-generation sequencing platform. By assembling contigs and scaffolds, we predicted and analyzed the GO and KEGG terms for potential genes and proteins involved in the synthesis of polyunsaturated fatty acids. These data could yield novel insights into the molecular mechanisms of *Thraustochytriidae* sp. and serve in developing innovative strategies for promoting PUFA (including DHA) production.

## Figures and Tables

**Table 1:** Summary of clean data assembly for *Thraustochytriidae* sp. SZU445.

Sample Name	Seq Type (#)	Total Number (#)	Total Length (Mb)	N50 Length (Mb)	N90 Length (Mb)	Max Length (Mb)	Min Length (Mb)	Gap Number (Mb)	GC Content (%)
SZU445	Scaffold	25	61.97	5.98	2.41	13.75	0.0054	0.091	45.04
SZU445	Contig	54	61.88	2.55	1.39	4.00	0.0054	-	45.04

Seq Type (#): Sequence type (Scaffold, Contig). Total number (#): Total number of Contig or Scaffold. Total length (Mb): Total length of assembly results. N50 Length (Mb): The N50 length is used to determine the assembly continuity; the higher the better. N50 is a weighted median statistic that 50% of the total length is contained in transcripts that are equal to or larger than this value. N90 length (Mb): similar to N50 length. Max length (Mb): max length of scaffold or contig. Min length (Mb): min length of scaffold or contig. Gap number (Mb): number of gaps in the sequence. GC content (%): the percentage of G and C bases in the assembly result sequence.

**Table 2:** The Genetic component of *Thraustochytriidae* sp. SZU445.

Sample Name (#)	Type (#)	Total Number (#)	Total Length (bp)	Average Length (bp)	Length/Genome Length (%)
SZU445	Gene Stat	14,145	26,947,341	1905.08	43.48
	Exons Stat	18,768	25,518,500	1359.68	41.18
	CDS Stat	14,145	25,518,500	1804.07	41.18
	Intron Stat	4623	1,428,841	309.07	2.31

**Table 3:** Noncoding RNA statistics of *Thraustochytriidae* sp. SZU445.

Sample Name (#)	Type	Copy#	Avg_Len	Total_Len	% in Genome
	tRNA	493	77.81	38,362	0.0619
SZU445	rRNA	235	1683.92	395,723	0.6385
	snRNA	77	70.15	5402	0.0087

Type: ncRNA type. Copy: The number of ncRNA type copies. Avg_Len: The average length of ncRNA. Total_Len: The total length of ncRNA. % in Genome: ncRNA types as a percentage of the genome.

**Table 4:** Gene annotation results of *Thraustochytriidae* sp. SZU445 according to the database.

Total	CAZY	TCDB	IPR	SWISS-PROT	GO	KEGG	KOG	COG	P450	TF	EKPD	NOG	CARD	CWDE	NR	DBCAN	PHI	PHOSPHATASE	Overall
14,145	34 (0.24%)	232 (1.64%)	9707 (68.62%)	2122 (15%)	7255 (51.29%)	1625 (11.48%)	1866 (13.19%)	1324 (9.36%)	740 (5.23%)	364 (2.57%)	369 (2.60%)	3550 (25.09%)	7 (0.04%)	1 (0.7%)	2629 (18.58%)	207 (1.46%)	412 (2.91%)	85 (0.60%)	9852 (69.65%)

CAZY: Carbohydrate-Active enZYmes Database. TCDB: Transporter Classification Database. IPR: InterPro Database. GO: Gene Ontology Database. KEGG: Kyoto Encyclopedia of Genes and Genomes. KOG: EuKaryotic Orthologous Groups. COG: Clusters of Orthologous Groups. P450: Fungal Cytochrome P450 Database. TF: Transcription Factor database. EKPD: Eukaryotic Protein Kinases and Protein Phosphatases. NOG: Evolutionary genealogy of genes: Non-supervised Orthologous Groups. CARD: The Comprehensive Antibiotic Resistance Database. CWDE: Cell Wall Degrading Enzyme. NR: Non-Redundant Protein Database. DBCAN: a web server and Database for automated Carbohydrate-active enzyme ANnotation. PHI: Pathogen–Host Interactions. PHOSPHATASE: a phosphatases database of EKPD.

**Table 5:** Enzymes involved in fatty acid biosynthesis and metabolism identified by annotation of the *Thraustochytriidae* sp. SZU445 genome.

Enzyme	EC Number	Number of Transcripts
**Fatty Acid Desaturation and Elongation**		
delta7-sterol 5-desaturase	1.14.19.20	1
sphingolipid 8-(E)-desaturase	1.14.19.18	1
sphingolipid 4-desaturase	1.14.19.17 1.14.18.5	1
aldehyde dehydrogenase (NAD+)	1.2.1.3	4
17beta-estradiol 17-dehydrogenase	1.1.1.62 1.1.1.330	1
acyl-CoA dehydrogenase	1.3.8.7	145
glycerol-3-phosphate dehydrogenase	1.1.5.3	26
S-(hydroxymethyl)glutathione dehydrogenase/alcohol dehydrogenase	1.1.1.284 1.1.1.1	2
glycerol-3-phosphate dehydrogenase	1.1.5.3	26
glutaryl-CoA dehydrogenase	1.3.8.6	5
glycerol-3-phosphate dehydrogenase (NAD+)	1.1.1.8	1
alcohol dehydrogenase (NADP+)	1.1.1.2	9
aldehyde dehydrogenase family 7 member A1	1.2.1.31 1.2.1.8 1.2.1.3	2
3-hydroxyacyl-CoA dehydrogenase	1.1.1.35	29
glycerol 2-dehydrogenase (NADP+)	1.1.1.156	1
S-(hydroxymethyl)glutathione dehydrogenase/alcohol dehydrogenase	1.1.1.284 1.1.1.1	2
17beta-estradiol 17-dehydrogenase/very-long-chain 3-oxoacyl-CoA reductase	1.1.1.62 1.1.1.330	1
delta14-sterol reductase	1.3.1.70	1
**Fatty Acid Biosynthesis**		
acetyl-CoA acyltransferase 2	2.3.1.16	2
acetyl-CoA acyltransferase 1	2.3.1.16	2
hydroxymethylglutaryl-CoA synthase	2.3.3.10	6
fatty acid synthase subunit alpha	2.3.1.86	2
3-oxoacyl-[acyl-carrier-protein] synthase II	2.3.1.179	1
acetyl-CoA carboxylase/biotin carboxylase 1	6.4.1.2 6.3.4.14 2.1.3.15	1

**Table 6:** The National Center for Biotechnology Information (NCBI) accession numbers of the reference strains for the phylogenetic analysis.

Reference Strains	NCBI Accession
*Aurantiochytrium* sp. HS399	MH319338.1
*Aurantiochytrium* sp. ST-2012	JQ982490.1
*Aurantiochytrium* sp. LY-2012	JX847377.1
*Schizochytrium* sp. SH104	KX379459.1
*Thraustochytriidae* sp. NIOS-1	AY705769.1

## Supplementary Materials

The default parameters of Infranal software are available online at https://www.mdpi.com/1660-3397/18/2/118/s1.
