# Supplementary material for: Genome Sequencing and Analysis of Thraustochytriidae sp. SZU445 Provides Novel Insights into the Polyunsaturated Fatty Acid Biosynthesis Pathway
Source: Mar Drugs. 2020 Feb 18;18(2):118. doi: 10.3390/md18020118 (PMC7073664; doi:10.3390/md18020118)
Supplement: Supplementary file 1 [file marinedrugs-18-00118-s001.pdf]

Supplementary Material

The default parameters of Infranal software：

Usage: cmsearch [options] <cmfile> <seqdb>

Basic options:

-h : show brief help on version and usage

-g : configure CM for glocal alignment [default: local]

-Z <x> : set search space size in *Mb* to <x> for E-value calculations (x>0)

--devhelp : show list of otherwise hidden developer/expert options

Options directing output:

-o <f> : direct output to file <f>, not stdout

-A <f> : save multiple alignment of all significant hits to file <s>

--tblout <f> : save parseable table of hits to file <s>

--acc : prefer accessions over names in output

--noali : don't output alignments, so output is smaller

--notextw : unlimit ASCII text output line width

--textw <n> : set max width of ASCII text output lines [120] (n>=120)

--verbose : report extra information; mainly useful for debugging

Options controlling reporting thresholds:

-E <x> : report sequences <= this E-value threshold in output [10.0] (x>0)

-T <x> : report sequences >= this score threshold in output

Options controlling inclusion (significance) thresholds:

--incE <x> : consider sequences <= this E-value threshold as significant [0.01]

--incT <x> : consider sequences >= this score threshold as significant

Options controlling model-specific reporting thresholds:

--cut_ga : use CM's GA gathering cutoffs as reporting thresholds

--cut_nc : use CM's NC noise cutoffs as reporting thresholds

--cut_tc : use CM's TC trusted cutoffs as reporting thresholds

Options controlling acceleration heuristics*:

--max : turn all heuristic filters off (slow)

--nohmm : skip all HMM filter stages, use only CM (slow)

--mid : skip first two HMM filter stages (SSV & Vit)

--default : default: run searc) to search for RNA sequences in genomic data, sRNA, miRNA and snRNA was predicted in compared with Rfam database (Version: 9.1, parameter settings: －p blastn －W 7 －e 1 －v 10000 －b 10000 －m 8 －i subfile －o *.blast.m8
